# Venous thromboembolism in lung transplant recipients: timing and clinical impact, a 10-year cohort analysis

**DOI:** 10.1016/j.jhlto.2025.100458

**Published:** 2025-12-04

**Authors:** Dieuwertje Ruigrok, Lucie E. Boersma, Jon Admiraal, Ares M. Rasoul, Nuray Kusadasi, Sue A. Braithwaite, Linda M. de Heer, Bart Luijk, Rob Schönwetter, Marco C. Post

**Affiliations:** a Department of Pulmonary Diseases, University Medical Center Utrecht, Utrecht, the Netherlands; b Department of Anesthesiology, University Medical Center Utrecht, Utrecht, the Netherlands; c Department of Intensive Care Medicine, University Medical Center Utrecht, Utrecht, the Netherlands; d Department of Cardiothoracic Surgery, University Medical Center Utrecht, Utrecht, the Netherlands; e Department of Pulmonary Diseases, St. Antonius Hospital, Nieuwegein, the Netherlands; f Department of Cardiology, University Medical Center Utrecht, Utrecht, the Netherlands; g Department of Cardiology, St. Antonius Hospital, Nieuwegein, the Netherlands

**Keywords:** Lung transplantation, Venous thromboembolism, Pulmonary embolism, Survival

## Abstract

**Introduction:**

The burden of venous thromboembolism (VTE) in lung transplant (LTx) recipients is not fully elucidated despite its frequent occurrence. We report the incidence of VTE and characteristics of VTE in a large LTx cohort, assess severity using risk stratification algorithms, and explore associations with graft survival and chronic lung allograft dysfunction (CLAD).

**Methods:**

A retrospective cohort analysis was performed on LTx recipients transplanted between July 2012 and August 2022 in our center. Primary outcome was incidence of VTE. Three groups were compared as follows: patients with early VTE (i.e*.,* within the first 6 months after LTx), late VTE (i.e*.,* beyond the first 6 months after LTx), and no VTE.

**Results:**

About 94/286 patients (33%) were diagnosed with VTE (median follow-up of 4.3 years): 67/94 (71%) early VTE and 27/94 (29%) late VTE. 66/94 (70%) of VTE events were pulmonary embolism (PE). In the early VTE group, none of the PE cases were classified as intermediate-high risk PE and 1 patient had high-risk (fatal) PE. Graft survival was not different between groups; median CLAD-free survival was shorter in patients with late VTE (6.2 years) compared to patients without VTE during follow-up (9.8 years) (*p* = 0.044).

**Conclusions:**

Thirty-three percent of LTx recipients developed VTE, 71% within the first 6 months after LTx. Seventy percent of cases were acute PE, but fatal or severe nonfatal PE were rare. No effect of VTE on graft survival was seen; CLAD-free survival was shorter in patients with late VTE compared to patients without VTE.

Lung transplantation (LTx) is a life-saving treatment in eligible patients with end-stage lung failure.^1^ Despite major advancements in peri- and postoperative care, LTx is associated with major morbidity and mortality. Median graft survival in Europe is 6.8 years.^2^ While chronic lung allograft dysfunction (CLAD), infections, and malignancies are well-established complications limiting survival,^3^, ^4^ venous thromboembolism (VTE) receives less attention despite reported incidences after LTx ranging from 6% to 44%.^5^, ^6^, ^7^, ^8^, ^9^, ^10^, ^11^ This wide range reflects differences in study design, time frames analyzed, and screening practices.

In the general population, VTE and especially hospital-associated VTE (i.e*.,* deep vein thrombosis (DVT) and/or pulmonary embolism (PE) occurring during or within 90 days of hospital admission) is linked to increased mortality, prolonged hospital length of stay (LOS), and long-term complications.^12^, ^13^ Its burden in LTx recipients remains insufficiently understood. Most studies report that early postoperative VTE is associated with longer LOS.^6^, ^8^, ^9^ These early VTE might reflect a patient population with increased risk following LTx and therefore more susceptible to complications such as VTE. Furthermore, publications specifically reporting on the severity of VTE post-LTx, especially using PE risk scores (such as the simplified Pulmonary Embolism Severity Index [sPESI])^14^ and the acute PE risk stratification algorithm,^15^ are absent.

Regarding long-term effects, conflicting results have been reported on the relation between VTE after LTx and survival.^7^, ^9^, ^11^ In addition, data on any association between VTE and CLAD are scarce, indicating no association with time-to-CLAD.^16^

The aim of this retrospective analysis is to report the incidence of VTE and characteristics of VTE in a large LTx cohort, to assess severity using risk stratification algorithms, and to explore associations with graft survival and CLAD.

## Methods

### Study subjects

In this retrospective analysis, all consecutive patients receiving a lung transplant between July 2012 and August 2022 in the University Medical Center Utrecht, Utrecht, the Netherlands, were analyzed, with follow-up continued until January 2025 (censor date 1-1-2025). After the initial hospital stay for LTx, patients were followed in either the University Medical Center Utrecht or in St. Antonius Hospital Nieuwegein (these centers work together as one transplant clinic). Patients with incomplete follow-up, for example, because they transferred to another transplant center in the Netherlands or abroad, were excluded.

Standard pharmacological thromboprophylaxis was given to patients during their entire postoperative hospital stay (dalteparin 2500 IE or 5000 IE once daily subcutaneously), unless an indication for therapeutic anticoagulation was present. Thromboprophylaxis was stopped at hospital discharge, and no mechanical thromboprophylaxis was used.

The study did not fall within the scope of the Medical Research Involving Human Subjects Act, since a retrospective analysis was performed and subjects were not subjected to additional investigations. This was confirmed by the Medical Ethics Review Committee of the University Medical Center Utrecht (21/328), and it was exempt from full review and informed consent.

### Study design and statistical analysis

VTE events were scored, including the type of VTE (PE, DVT of either upper or lower extremity), and in case of PE also the European Society for Cardiology (ESC)/European Respiratory Society (ERS) risk classification^15^ was assigned retrospectively (and repeated if already stated at the time of diagnosis). Ventricular diameters were assessed on axial transverse computed tomography (CT) images, measuring the maximal distance between the ventricular endocardium and the interventricular septum.^17^ Right ventricular (RV) dysfunction on imaging was defined as right ventricle/left ventricle (RV/LV) ratio on CT ≥1.0 according to the ESC/ERS guideline,^15^ and was determined retrospectively in all cases by a single observer (DR) with training and experience in determining RV/LV ratio on CT.

VTE events were classified as early, i.e*.,* diagnosed within 6 months after LTx, or late, i.e*.,* diagnosed beyond 6 months after LTx. In our clinical practice, routine CT scans (not as part of any screening for VTE) are performed around 3 to 5 months post-LTx, and these CT scans reveal incidental PE in a number of patients. We consider such findings to be potentially related to the transplant procedure itself. To ensure these events are captured within the early VTE group, we chose a 6-month cutoff.

Synchronous PE and DVT are counted as one event. Recurrent VTE was defined as a secondary event diagnosed after at least 3 months of adequate anticoagulation.

Graft survival and CLAD analysis were performed conditional on 90-day survival (i.e*.,* all patients who died within 90 days after LTx were excluded). CLAD was defined according to the most recent consensus report of the International Society for Heart and Lung Transplantation (ISHLT), based on a 20% persistent decline in measured forced expiratory volume in 1 s (FEV1) from baseline FEV1, not explained by other conditions such as mechanical factors.^3^

Primary outcome of this study was incidence of VTE. Secondary outcomes consisted of characteristics of the VTE, graft survival and CLAD. Three groups were compared as follows: patients with early VTE, late VTE, and no VTE.

Data are presented as mean (standard deviation), median (interquartile range [IQR]), or number of patients (%). Missing data were not imputed. Normal distribution was tested by using D′Agostino-Pearson omnibus normality test; log-transformation was performed when distribution was not normal. Differences regarding continuous data (3 groups: no VTE, early VTE, and late VTE) were tested using Kruskal-Wallis test with Dunn’s multiple comparisons test. Differences regarding categorical data were tested using Chi-square test or Fisher’s exact test.

Univariate and multivariate logistic regression analysis were performed to analyze factors associated with early VTE. Multivariate analysis was performed on parameters with *p* < 0.100 in univariate analysis.

Kaplan-Meier survival curves were used to analyze graft survival and CLAD-free survival; comparison of groups was performed with the log-rank test.

Values of *p* < 0.05 were considered to reflect statistical significance. Statistical analysis was performed using GraphPad Prism version 10 (GraphPad Software Inc., La Jolla, CA) and IBM SPSS Statistics version 29.

## Results

**Figure 1:**
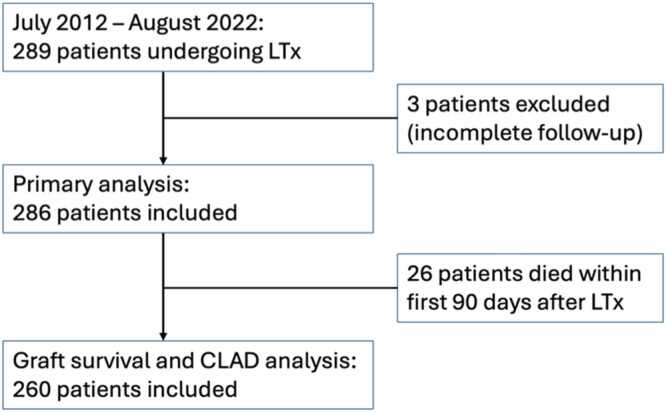
Flow chart of included patients. CLAD, chronic lung allograft dysfunction; LTx, lung transplantation

Between July 2012 and August 2022, 289 patients received a lung transplant in our center. Three patients were excluded because of incomplete follow-up (follow-up was continued in other transplant centers). The remaining 286 patients were analyzed in the primary analysis on the occurrence of VTE and its characteristics. Two hundred and sixty patients were included in the secondary analysis on graft survival and CLAD, conditional on 90-day survival (28 patients died within the first 90 days after LTx) (Figure 1).

Baseline characteristics of these 286 patients are described in Table 1: a large majority of transplants consists of bilateral LTx, and the predominant indication for LTx in our center is restrictive lung disease (46.5% of all transplants).

**Table 1:** Baseline Characteristics

	286 LTx Recipients
Age at LTx (y)	57 (48-61)
Female	135 (47.2%)
BMI (kg/m^2^)	23.5 (21.1-26.6)
*Type of transplant*	
Bilateral	260 (90.9%)
Left	10 (3.5%)
Right	16 (5.6%)
*Transplant indication*	
Obstructive lung disease	90 (31.5%)
Disease of pulmonary circulation	7 (2.4%)
Suppurative lung disease	48 (16.8%)
Restrictive lung disease	133 (46.5%)
Retransplant	8 (2.8%)
*Use of ECC*	
ECMO pre-LTx (bridging)	13 (4.5%)
ECC intraoperative (ECMO or CPB)	193 (67.5%)
Extended ECMO post-LTx	97 (33.9%)
*Hospital LOS*	
Survival to discharge	262 (91.6%)
Duration ICU admission in survivors (days)	8 (4-16)
Total LOS in survivors (days)	30 (23-47)
*Graft survival and CLAD-free survival (n = 260)*	
Median graft survival, conditional on 90-day survival (days)	3437 (95% CI 2887-3986)
Median CLAD-free survival, conditional on 90-day survival (days)	3294 (95% CI 2746-3842)

Data presented as median (IQR), median (95% confidence interval) or number of patients (%)

BMI, body mass index; CI, confidence interval; CLAD, chronic lung allograft dysfunction; CPB, cardiopulmonary bypass; ECC, extracorporeal circulation; ECMO, extracorporeal membrane oxygenation; ICU, intensive care unit; LOS, length of stay; LTx, lung transplantation

In total, 94 patients (33%) were diagnosed with a primary VTE during follow-up after LTx (median follow-up duration 4.3 years [IQR 2.3-7.6 years]) (Table 2). Of these 94 patients, 67 events (71% of all primary VTE events) were diagnosed early after LTx (within the first 6 months after LTx), and 27 events (29% of all primary VTE events) were diagnosed late after LTx (beyond the first 6 months). Exactly 48/67 (72%) of early VTE were diagnosed during the initial hospital stay for LTx. Fifteen patients had a recurrent VTE during follow-up. Seven patients with synchronous DVT and PE are described in detail in supplemental table A.

**Table 2:** VTE Characteristics

	Early VTE (n = 67)	Late VTE (n = 27)
Time to VTE diagnosis (days)	10 (6-61)	719 (390-2366)
*Type of VTE*		
PE	43 (64.2%)	16 (59.3%)
DVT lower extremity	8 (11.9%)	5 (18.5%)
DVT upper extremity	14 (20.9%)	1 (3.7%)
PE + DVT lower extremity	1 (1.5%)	5 (18.5%)
PE + DVT upper extremity	1 (1.5%)	0 (0.0%)
*Characteristics of PE*		
Incidental PE	5 (11%)	0 (0.0%)
Locations of PE (n = 62 with available CT) Central Segmental Subsegmental	n = 42 with available CT*	n = 20 with available CT**
	9 (21%)	7 (35%)
	21 (50%)	11 (55%)
	12 (29%)	2 (10%)
RV/LV ratio >1.0	1 (2.4%)*	3 (15.0%)**
*PE risk stratification (n = 66)*		
Low risk PE	4 (8.9%)	8 (38.1%)
Intermediate-low risk PE	37 (82.2%)	10 (47.6%)
Intermediate-high risk PE	0 (0.0%)	2 (9.5%)
High-risk PE	1 (2.2%)	0 (0.0%)
Unknown risk stratification	3 (6.7%)	1 (4.8%)

Data presented as median (IQR) or number of patients (%)

CT, computed tomography; DVT, deep vein thrombosis; LV, left ventricle; PE, pulmonary embolism; RV, right ventricle; VTE, venous thromboembolism

* In the early VTE group, PE with or without DVT was diagnosed with CTA in 43/45 PE patients; CTA was available for analysis in 42 patients

** In the late VTE group, PE with or without DVT was diagnosed with CTA in 20/21 PE patients, all 20 CTA were available for analysis

Table 2 describes the characteristics of both early and late VTE. In both groups, PE was the most prevalent manifestation of VTE (present in 66/94 cases (70%)).

Regarding the severity of PE, signs of RV dysfunction on CT imaging (defined as RV/LV ratio ≥1.0) were present in 4/62 (6%), and risk stratification scores were low risk in 12/66 (18%) and intermediate-low risk in 47/66 (71%). None of the early PE cases was classified as intermediate-high risk, compared to 2/21 (9.5%) of the late PE cases. One case (early VTE) was scored as high-risk acute PE (defined as acute PE associated with hemodynamic instability). However, in this patient, death was already imminent even without acute PE. None of the VTE cases, neither early nor late, received interventional or surgical treatment, all cases were treated with conventional anticoagulation.

Regarding possible deaths due to occult acute PE: 118 patients died during follow-up. Review of causes of death and autopsy findings (performed in 31 patients) resulted in 1 death due to occult massive PE.

About 55/94 patients (59%) were using pharmacological thromboprophylaxis at the time of VTE diagnosis. A comparison between VTE in patients with or without thromboprophylaxis is detailed in supplemental table B. PE diagnosed when not using thromboprophylaxis were classified as low-risk PE in 11/33 (33%), whereas those occurring while using thromboprophylaxis were intermediate-low risk PE in 28/33 (85%).

**Figure 2:**
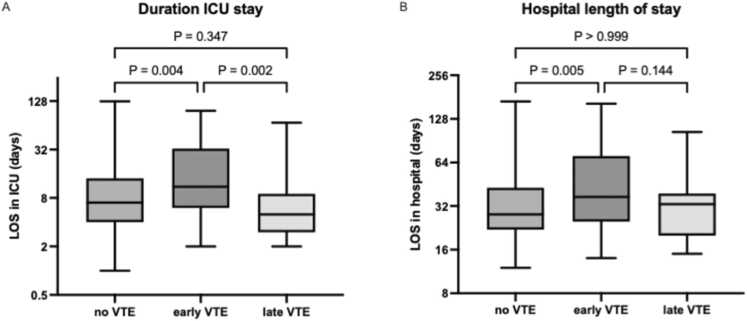
A: comparison of duration of ICU stay between groups; Dunn’s multiple comparisons test performed. B: comparison of hospital length of stay between groups; Dunn’s multiple comparisons test performed. ICU, intensive care unit; LOS, length of stay; VTE, venous thromboembolism

Comparison of patients with early VTE, late VTE, and no VTE indicated early VTE was significantly more prevalent in patients with restrictive lung disease as the indication for LTx (61.2%), compared to nonrestrictive lung disease indications (38.8%) (Table 3). In addition, use of extended venovenous extracorporeal membrane oxygenation (VV-ECMO) after LTx was more frequent in patients with early VTE (28.4%), as was their LOS in the intensive care unit (ICU) (median 11 days) and total hospital LOS (median 37 days) longer (Figure 2). The proportion of in-hospital deaths was similar in all groups; 24 patients died during the initial hospital stay following LTx; their causes of death are detailed in supplemental table C.

**Table 3:** Comparison of Patients Without VTE, With Early VTE, and Late VTE

	No VTE (n = 192)	Early VTE (n = 67)	Late VTE (n = 27)	*p* value
Age at LTx (y)	57 (47-62)	58 (52-62)	54 (47-61)	0.674
Female	92 (47.9%)	26 (38.8%)	17 (63.0%)	0.102
BMI (kg/m^2^)	23.2 (21.0-26.2)	24.9 (21.4-27.5)	23.1 (20.5-27.8)	0.210
*Type of transplant*				
Bilateral	175 (91.1%)	60 (89.6%)	25 (92.6%)	
Unilateral left or right	17 (8.9%)	7 (10.4%)	2 (7.4%)	
Uni- vs bilateral				0.953
DBD vs DCD/euthanasia donor	103 (53.6%) vs 89 (46.4%)	39 (58.2%) vs 28 (41.8%)	19 (70.4%) vs 8 (29.6%)	0.242
EVLP	22 (11.5%)	9 (13.4%)	0 (0.0%)	0.148
*Transplant indication*				
Obstructive lung disease	66 (34.4%)	13 (19.4%)	11 (40.7%)	
Disease of pulmonary circulation	6 (3.1%)	1 (1.5%)	0 (0.0%)	
Suppurative lung disease	35 (18.2%)	8 (11.9%)	5 (18.5%)	
Restrictive lung disease	81 (42.2%)	41 (61.2%)	11 (40.7%)	
Retransplant	4 (2.1%)	4 (6.0%)	0 (0.0%)	
Restrictive vs nonrestrictive lung disease	81 (42.2%) vs 111 (57.8%)	41 (61.2%) vs 26 (38.8%)	11 (40.7%) vs 16 (59.3%)	0.022*
*Use of ECC*				
ECMO pre-LTx (bridging)	11 (5.7%)	2 (3.0%)	0 (0.0%)	0.486
Intraoperative CPB	20 (10.4%)	4 (6.0%)	4 (14.8%)	0.375
Intraoperative VA-ECMO	111 (57.8%)	43 (64.2%)	12 (44.4%)	0.213
Extended ECMO post-LTx	61 (31.8%)	31 (46.3%)	5 (18.5%)	0.020*
Extended VV-ECMO	26 (13.5%)	19 (28.4%)	2 (7.4%)	0.008*
Extended VA-ECMO	35 (18.2%)	12 (17.9%)	3 (11.1%)	0.656
*Early mortality and hospital length of stay (LOS)*				
Survival to discharge	172 (89.6%)	63 (94.0%)	27 (100%)	0.165
Duration ICU admission in survivors (days)	7 (4-14)	11 (6-33)	5 (3-9)	<0.001*
Total LOS in survivors (days)	28 (22-43)	37 (25-71)	33 (20-39)	0.006*

BMI, body mass index; CPB, cardiopulmonary bypass; DBD, donation after brain death; DCD, donation after circulatory death; ECC, extracorporeal circulation; ECMO, extracorporeal membrane oxygenation; EVLP, ex vivo lung perfusion; ICU, intensive care unit; LOS, length of stay; LTx, lung transplantation; VA, venoarterial; VTE, venous thromboembolism; VV, venovenous. Data presented as median (IQR) or number of patients (%). Statistical tests used: Kruskal-Wallis test, Chi-square test, Fisher’s exact test. Statistical significance indicated with an *.

Multivariate logistic regression analysis confirmed associations between early VTE and restrictive lung disease as the indication for LTx (odds ratio (OR) 2.09, 95% confidence interval (CI) 1.14 to 3.82, *p* 0.017) and retransplantation (OR 5.19, 95% CI 1.15 to 23.41, *p* 0.032)(Table 4). Additional analysis showed that patients with restrictive lung disease were significantly older (median 59 years, IQR 54 to 62) compared to those with nonrestrictive lung disease (median 54 years, IQR 38 to 60) (*p* < 0.001).

**Table 4:** Logistic Regression Analysis on Association With Early VTE

	Univariate analysis: OR (95% CI), *p* value	Multivariate analysis: OR (95% CI), *p* value
Restrictive lung disease as indication for LTx (compared to nonrestrictive lung disease)	OR 2.18 (1.24-3.81), *p* 0.006	OR 2.09 (1.14-3.82), *p* 0.017
Retransplant	OR 3.41 (0.83-14.04), *p* 0.089	OR 5.19 (1.15-23.41), *p* 0.032
BMI ≥23.5 kg/m^2^	OR 1.96 (1.12-3.44), *p* 0.019	
Age at LTx ≥57 y	OR 1.55 (0.89-2.70), *p* 0.122	
Extended/postoperative VV-ECMO	OR 2.70 (1.39-5.24), *p* 0.003	OR 1.98 (0.97-4.01), *p* 0.058
Duration ICU stay ≥8 days	OR 2.42 (1.36-4.33), *p* 0.003	OR 1.82 (0.97-4.00), *p* 0.060
LOS ≥30 days	OR 2.04 (1.16-3.57), *p* 0.013	

BMI, body mass index; CI, confidence interval; ICU, intensive care unit; LOS, length of stay; LTx, lung transplantation; OR, odds ratio; VTE, venous thromboembolism; VV-ECMO, venovenous extracorporeal membrane oxygenation.

**Figure 3:**
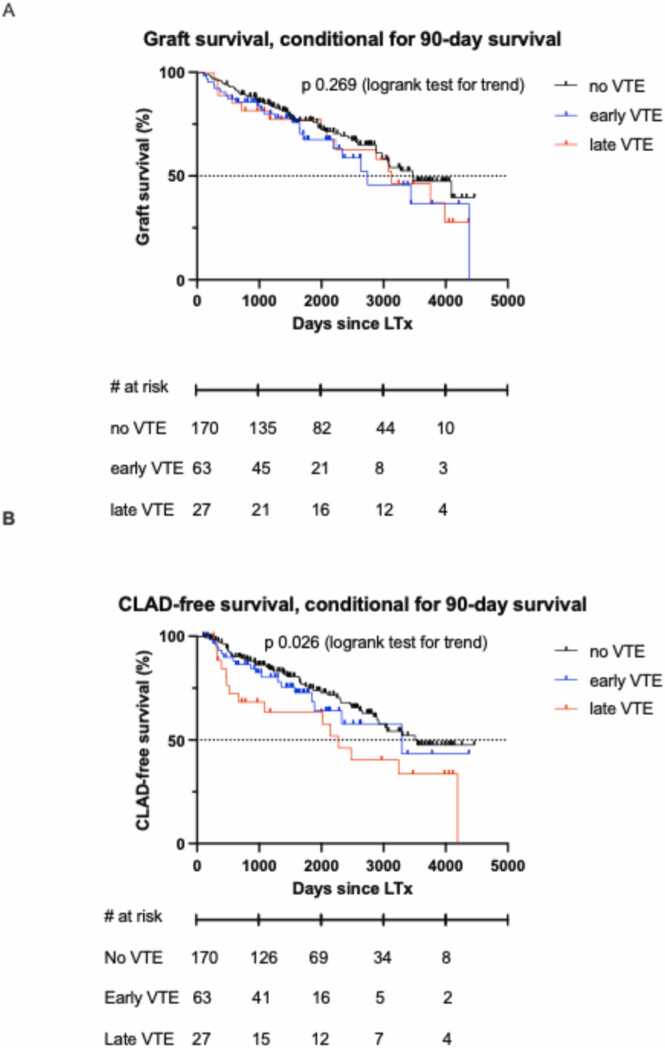
A: graft survival, conditional on 90-day survival. B: CLAD-free survival, conditional on 90-day survival. CLAD, chronic lung allograft dysfunction; LTx, lung transplantation; VTE, venous thromboembolism.

Graft survival, conditional on 90-day survival, was not different between groups: 9.5 years in patients without VTE during follow-up, 7.5 years in patients with early VTE and 8.6 years in patients with late VTE (*p* 0.269). However, median CLAD-free survival was significantly different between groups (Figure 3): 6.2 years in patients with late VTE, 9.0 years in patients with early VTE and 9.8 years in patients without VTE. Additional testing revealed late VTE was associated with significantly lower CLAD-free survival compared to patients without VTE (*p* 0.044 [logrank test, corrected for multiple testing]).

## Discussion

In this cohort of 286 LTx recipients, 33% of patients developed VTE during follow-up: 71% within the first 6 months after LTx (early VTE) and 29% thereafter (late VTE). Acute PE accounted for 70% of VTE events, with RV dysfunction present in only 6.5%, notably lower than in the general population. Early VTE occurred more frequently in patients with a restrictive lung disease indication for LTx and retransplants. Graft survival did not differ between patients with or without VTE (either early or late). However, late VTE was associated with reduced CLAD-free survival compared to no VTE.

The incidence of VTE in our analysis is consistent with previous reports,^5^, ^6^, ^7^, ^8^, ^9^, ^10^, ^11^ although the proportion of PE is strikingly higher in our cohort. Notably, 33% of LTx recipients developed VTE despite routine inpatient pharmacological thromboprophylaxis. Seventeen percent were diagnosed with VTE during the initial hospital stay, markedly higher than the 2.2% reported in a large prospective study of high-risk internal medicine inpatients receiving similar thromboprophylaxis.^18^

Several factors contribute to the prothrombotic state in LTx recipients. Early post-LTx risks include reduced mobility, recent surgery, recent major bleeding, anemia, acute infections, and indwelling venous catheters.^18^, ^19^ Later, additional VTE risk factors emerge in this population, such as microalbuminuria, chronic kidney disease,^20^, ^21^ cancer,^22^ and increasing age. Immunosuppressive medications, including glucocorticoids^23^ and mechanistic target of rapamycin (mTOR) inhibitors,^24^ further increase VTE risk. A history of VTE (as a risk factor) was present in 7% of affected patients in our cohort.

This study is the first to provide a detailed analysis of PE severity in LTx recipients. PE-related mortality is primarily driven by acute RV pressure overload, which can lead to RV failure and death if this sequence of events is not stopped by unloading the RV. Risk stratification is essential to identify hemodynamically stable patients with RV dysfunction, who are at increased risk of adverse outcomes. All PE cases (with or without concurrent DVT) were classified using the 2019 ESC/ERS risk stratification algorithm,^15^ with 89% categorized as low or intermediate-low risk acute PE. Notably, in the early PE group, RV dysfunction was absent in all but 1 patient, whose high-risk PE coincided with imminent death from other reasons. This contrasts with acute PE in the general population and raises questions about the origin and clinical relevance of early PE in this setting.

Some pulmonary thrombi may originate from the donor lungs, i.e*.,* related to the donor lung procurement, as thrombi are commonly observed during the retrograde flush in donor lung procurement. A study showing mismatched perfusion defects on routine VQ scan, with anticoagulation withheld in 80% of cases, reported no clinical signs of PE or long-term impact on survival or time-to-CLAD, supporting the possibility of donor-derived thrombi in select early PE cases.^16^ However, this remains speculative, and we were unable to objectively verify or quantify the number of potentially donor-derived thrombi.

Many early PE cases were diagnosed in the ICU setting, often alongside other causes of respiratory compromise. Imaging was typically guided by clinical judgment rather than clinical prediction rules and D-dimer testing, suggesting that some early PE may have been incidental or clinically insignificant, possibly donor-derived or subsegmental in a setting where it is unknown whether the symptoms are to be attributed to these subsegmental PE. In the early VTE group, 29% of PE were subsegmental, compared to 10% in the late VTE group. The low incidence of RV dysfunction, even in patients recovering from severe pretransplant RV strain, further questions the clinical relevance of these early PE events.

These subsegmental PE and potentially incidental or clinically insignificant PE likely account for the high proportion of PE among all VTE cases. This effect may have been further amplified by the low clinical threshold for performing CTA in patients with respiratory compromise not otherwise explained.

Regardless of the attributed clinical significance, all cases of PE in our cohort were treated accordingly with anticoagulants, in accordance with international guidelines based on the adjudicated risk for recurrent or progressive VTE if not anticoagulated and low cardiopulmonary reserve.^25^ Although we did not formally analyze bleeding complications, major bleeding complications related to anticoagulation for VTE were infrequent in our cohort. Based on our findings, we would not recommend routine screening for VTE or extended thromboprophylaxis early after LTx. While Moneke et al suggest considering extended thromboprophylaxis in high-risk subgroups,^11^ 56% of VTE cases in our cohort occurred despite inpatient pharmacological thromboprophylaxis. This raises questions about the effectiveness of extended thromboprophylaxis in this setting. And the question remains whether all early VTE are equally relevant. This is in line with the 2018 American Society of Hematology (ASH) guidelines for prophylaxis in hospitalized medical patients, recommending against extending pharmacological thromboprophylaxis after discharge.^26^ Whether combining pharmacological and mechanical prophylaxis (using pneumatic compression devices or graduated compression stockings) in surgical LTx patients, as suggested by the 2019 ASH guidelines for VTE prevention in surgical hospitalized patients,^27^ is effective in preventing early VTE after LTx is unknown.

Despite all this, given the high number of VTE in LTx recipients, either early or late, a low threshold towards considering VTE is warranted in LTx recipients.

Early VTE was more frequent in patients with a restrictive lung disease indication for LTx and those undergoing retransplantation, likely reflecting a more compromised clinical status. This may also explain the longer ICU stays observed in patients with early VTE: it is unlikely that VTE alone prolonged ICU stay; rather, these patients were more vulnerable to VTE with higher overall complexity and the presence of central venous lines as important risk factors for VTE. Moreover, patients with restrictive lung disease were significantly older than those with nonrestrictive lung disease, a demographic factor that may contribute to an increased risk of VTE.

Regarding long-term outcomes after LTx and VTE, graft survival did not differ between patients with or without VTE. However, CLAD-free survival was lower in patients with late VTE compared to those without VTE. Based on our data, conclusions regarding causality between late VTE and shorter time-to-CLAD cannot be drawn. While causality cannot be established, some PE cases may have been diagnosed during the evaluation of declining pulmonary function before a formal CLAD diagnosis. Among 15 patients with late VTE who developed CLAD, the median interval between VTE and CLAD diagnosis was 91 days (IQR −181 to 561 days), indicating that nearly half occurred within 6 months before or after CLAD diagnosis. Acute PE may contribute to an obstructive pulmonary function decline, potentially influencing CLAD diagnosis. Additionally, PE could hypothetically increase CLAD risk due to the impaired vascular supply following LTx with loss of the bronchial circulation. This is supported by our (unpublished) observation that pulmonary infarction in the setting of acute PE appears relatively frequent in LTx recipients. This is plausible because of the compromised bronchial circulation after LTx, which plays an essential role in the pathophysiology of pulmonary infarction.^28^

A key strength of this study is the large sample size and the long and near-complete follow-up with only 3 patients excluded. Limitations include the retrospective design, with sPESI and risk stratification class assigned retrospectively. Although troponin data were missing for most PE cases, risk stratification was still feasible using sPESI and RV/LV ratio on CT, which, when RV/LV ratio was normal, limited risk class to intermediate-low risk at most regardless of troponin level.

The retrospective design precluded an in-depth analysis of the role of prolonged ECMO duration and VTE risk. The small number of patients with CLAD and late VTE in the Kaplan-Meier survival analysis, along with the lack of control for potential confounders, also limits interpretation.

In conclusion, 33% of LTx recipients developed VTE during follow-up. While most events were PE, fatal or severe nonfatal PE were rare, raising questions on the origin and clinical relevance of especially early PE. Given the high incidence of VTE after LTx, a low threshold for clinical suspicion of VTE is warranted. Further research is needed to evaluate the role of combined pharmacological and mechanical thromboprophylaxis early after LTx, and to explore a potential link between VTE and CLAD.

## Financial support

Not applicable.

## Disclosure statement

None.

## Declaration of Competing Interest

The authors declare that they have no known competing financial interests or personal relationships that could have appeared to influence the work reported in this paper.

## Footnotes

Appendix A Supplementary data associated with this article can be found in the online version at doi:10.1016/j.jhlto.2025.100458.
