# Supplementary material for: Venous thromboembolism in lung transplant recipients: timing and clinical impact, a 10-year cohort analysis
Source: JHLT Open. 2025 Dec 4;11:100458. doi: 10.1016/j.jhlto.2025.100458 (PMC12775926; doi:10.1016/j.jhlto.2025.100458)
Supplement: Supplementary file 1 — Supplementary material [file mmc1.docx]

Supplemental material

Table A: specific details of VTE cases with synchronous PE and DVT

| Demographics | Perioperative course | VTE diagnosis and treatment | Outcome |
| --- | --- | --- | --- |
| Male, 35 yrs  Bilateral LTx for cystic fibrosis | ECMO bridge-to-LTx: no  Intraoperative VA-ECMO  Postoperative VV-ECMO (1 day)  ICU stay 6 days  Total LOS 46 days | 161 days post-LTx DVT upper extremity and PE  Diagnostic modality: ultrasound and CT-neck (upper part of thorax included)  PE risk stratification unknown  Risk factors: PICC in situ  No use of thromboprophylaxis at time of VTE diagnosis  Treatment: LMWH 3 months | CLAD: yes  Death: 7.5 years after LTx  Cause of death: COVID19 infection, MOF (complicated by recurrence of PE) |
| Male, 63 years  Right-sided LTx for COPD | ECMO bridge-to-LTx: no  No intraoperative CPB or VA-ECMO  Postoperative ECMO: no  ICU stay 2 days  Total LOS 25 days. | 109 days post-LTx DVT lower extremity and PE  Diagnostic modality: CTA  Central PE, RV/LV ratio < 1.0  sPESI 0  Risk stratification: low risk  No known provoking factors  No use of thromboprophylaxis at time of VTE diagnosis  Treatment: VKA, duration indeterminate | CLAD: yes  Death: 6.4 years after LTx  Cause of death: COVID19 infection |
| Female, 54 years  Bilateral LTx for COPD | ECMO bridge-to-LTx: no  No intraoperative CPB or VA-ECMO  Postoperative ECMO: no  ICU stay 3 days  Total LOS 15 days | 7.2 years post-LTx DVT lower extremity and PE  Diagnostic modality: CTA  Central PE, RV/LV ratio < 1.0  sPESI 3  Risk stratification: intermediate-low risk  No known provoking factors  No use of thromboprophylaxis at time of VTE diagnosis  Treatment: VKA, duration indeterminate | CLAD: yes  Death: 10.3 years after LTx  Cause of death: CLAD |
| Male, 52 years  Bilateral LTx for ILD in antisynthetase syndrome | ECMO bridge-to-LTx: no  Intraoperative CPB  Postoperative ECMO: no  ICU stay 6 days  Total LOS 27 days | 10.3 years post-LTx DVT lower extremity and PE  Diagnostic modality: CTA  Central PE, RV/LV ratio $\geq$ 1.0  sPESI 1  Risk stratification: intermediate-low risk  No known provoking factors  No use of thromboprophylaxis at time of VTE diagnosis  Treatment: DOAC, duration indeterminate | CLAD: no  Death: no (alive 11.3 years after LTx) |
| Female, 47 years  Bilateral LTX for FPF | ECMO bridge-to-LTx: no  Intraoperative VA-ECMO  Postoperative ECMO: no  ICU stay 3 days  Total LOS 17 days | 2.0 years post-LTx DVT lower extremity and PE  Diagnostic modality: CTA  Central PE, RV/LV ratio < 1.0  sPESI 0  Risk stratification: low risk  Provoking factors: recent immobilization  No use of thromboprophylaxis at time of VTE diagnosis  Treatment: DOAC, duration indeterminate | CLAD: no  Death: no (alive 9.5 years after LTx) |
| Male, 61 years  Bilateral LTx for IPF | ECMO bridge-to-LTx: no  Intraoperative VA-ECMO  Postoperative ECMO: no  ICU stay 2 days  Total LOS 17 days | 1.7 years post-LTx DVT lower extremity and PE  Diagnostic modality: CTA  Central PE, RV/LV ratio < 1.0  sPESI 2  Risk stratification: intermediate-low risk  No provoking factors  No use of thromboprophylaxis at time of VTE diagnosis  Treatment: VKA, duration indeterminate | CLAD: yes  Death: no (alive 8.4 years after LTx) |
| Male, 70 years  Bilateral LTx for IPF | ECMO bridge-to-LTx: no  No intraoperative CPB or VA-ECMO  Postoperative ECMO: no  ICU stay 5 days  Total LOS 21 days | 327 days post-LTx DVT lower extremity and PE  Diagnostic modality: CTA  Segmental PE, RV/LV ratio < 1.0  sPESI 0  Risk stratification: low risk  No provoking factors  No use of thromboprophylaxis at time of VTE diagnosis  Treatment: DOAC, duration indeterminate | CLAD: no  Death: no (alive 3.2 years after LTx) |

Abbreviations: VTE: venous thromboembolism; PE: pulmonary embolism; DVT: deep vein thrombosis; LTx: lung transplantation; ECMO: extracorporeal membrane oxygenation; VA: veno-arterial; VV: veno-venous; ICU: intensive care unit; LOS: length of stay; CT(A): computed tomography (angiography); PICC: peripheral inserted central catheter; LMWH: low-molecular-weight heparin; CLAD: chronic lung allograft dysfunction; MOF: multi-organ failure; COPD: chronic obstructive pulmonary diseases; CPB: cardiopulmonary bypass; RV/LV: right ventricular/left ventricular; sPESI: simplified pulmonary embolism severity index; VKA: vitamin K antagonist; ILD: interstitial lung disease; DOAC: direct oral anticoagulant; FPF: familial pulmonary fibrosis; IPF: idiopathic pulmonary fibrosis.

Table B: comparison of VTE in patients with or without thromboprophylaxis

|  | **With thromboprophylaxis (n = 55)** | **Without thromboprophylaxis (n = 39)** |
| --- | --- | --- |
| Age at LTx (years) | 56 (46-61) | 57 (52-62) |
| Female | 28 (51%) | 15 (38%) |
| BMI (kg/m^2^) | 24.2 (21.2-26.6) | 25.4 (21.3-28.4) |
| Time to VTE diagnosis (days) | 9 (5-25) | 224 (87-808) |
| *Type of VTE* | |  |
| PE | 33 | 26 |
| DVT lower extremity | 7 | 6 |
| DVT upper extremity | 15 | 0 |
| PE + DVT lower extremity | 0 | 6 |
| PE + DVT upper extremity | 0 | 1 |
| *PE risk stratification (n = 66)* | | |
| Low risk PE | 1 | 11 |
| Intermediate-low risk PE | 28 | 19 |
| Intermediate-high risk PE | 1 | 1 |
| High risk PE | 1 |  |
| Unknown risk stratification | 2 | 2 |

presented as median (IQR) or number of patients (%).

Abbreviations: VTE: venous thromboembolism; BMI: body mass index; PE: pulmonary embolism; DVT: deep vein thrombosis

Table C: causes of deaths among 24 patients who died during the initial hospital stay following LTx

| **Cause of in-hospital death** | **No VTE (20 patients)** | **Early VTE (4 patients)** |
| --- | --- | --- |
| Graft failure | 2 | 1 |
| Primary graft dysfunction | 1 | 1 |
| Infection | 6 |  |
| Technical complications | 3 |  |
| Multi-organ failure/refractory shock | 5 | 1 |
| CVA (haemorrhagic) | 2 |  |
| Infaust prognosis after a long and complicated ICU trajectory |  | 1 |
| Cardiac arrest potentially in the setting of severe aortic valve stenosis | 1 |  |

Data presented as absolute numbers.

Abbreviations: VTE: venous thromboembolism; CVA: cerebrovascular accident; ICU: intensive care unit
